# Cell Viability Assay of Chitosan-Modified Glass Ionomer Restorative Cements

**DOI:** 10.3390/jfb16120432

**Published:** 2025-11-24

**Authors:** Riaan Mulder, Suné Mulder-van Staden, Annette Olivier

**Affiliations:** 1 Department of Prosthodontics, University of the Western Cape, Bellville, Cape Town 7530, South Africa; 2 Oral Medicine, Periodontology and Implantology Department, The University of the Western Cape, Bellville, Cape Town 7530, South Africa; smuldervanstaden@uwc.ac.za; 3 Oral and Dental Research Laboratory, University of the Western Cape, Bellville, Cape Town 7530, South Africa; aolivier@uwc.ac.za

**Keywords:** chitosan, glass ionomer cement, cytocompatibility, 3T3 Balb/c fibroblast cells, DMEM diluent medium

## Abstract

**Purpose:** The present study evaluates the cytocompatibility of chitosan (CS)-modified glass ionomer cement (GIC) diluents for a Balb/c 3T3 fibroblast cell line. **Methods:** Three different commercially available hand-mix GIC materials were used in this experiment: Fuji IX GP, Ketac Universal, and Riva Self Cure. The diluents for cell viability tests were produced from DMEM exposed to sterile CS-modified glass ionomer material specimens for three different time periods (0–1, 1–7, and 7–21 days). The resultant diluents were exposed to a 3T3 fibroblast cell line using the indirect contact technique in 96-well plates. In order to assess the physical cell response, five material specimens (1 mm high and 3 mm in diameter) of each material (*n* = 45) were produced and 3T3 cells were seeded on the specimens. SEM evaluation of the cells was conducted. **Results:** All the Ketac Universal materials resulted in a decrease in cell viability on day 1. Fuji IX and the CS-modified GICs are the most consistent regarding cell viability. None of the CS-modified GICs exhibited improved cumulative cell biocompatibility. **Conclusion:** Two materials—Riva Self Cure modified with 5% and 10% CS—retained a decreased cell viability at day 21 compared to the viability of 3T3 cells exposed to the control DMEM.

## 1. Introduction

Glass ionomer cements (GICs) are multi-component materials consisting of a powder (fluoro-alumina-silicate glass) and a liquid component (polyacrylic acid) that must be mixed by a clinician in a prescribed ratio. The setting process of a conventional GIC is characterized by interactions between a weak acid and the glass powder upon mixing. The interactions of glass ionomer cements with living cells are important to the long-term health of pulpal tissue and the production of tertiary dentine. The favorable bioactive properties of GICs lie in their ability to form a physicochemical bond with the tooth structure [1]. This physicochemical bond is primarily caused by ion exchange and integration with the moist tooth structure [2]. The polyalkenoic acid portion of the liquid interacts with the powder to allow various metallic ions to be released from the glass particles to form a primary polysalt matrix [3] and subsequently, a siliceous hydrogel is left on the glass particle’s surface [4,5]. Therefore, the interaction of the liquid with the powder has an effect on the number of ions released and directly influences matrix formation. GICs continue to absorb water from moist dentine, allowing additional acid–base reactions to take place over time [6]. This absorbed moisture is essential for gradual maturation and increasing the compressive strength, the latter of which is caused by hydration of the silicate phase [7]. The anticariogenic properties of GICs due to the continuous release of fluoride [8,9] and other ions are advantageous for both sound and compromised tooth structures [10]. CS has not yet been added in powder form to the component of the GIC under investigation; it is generally incorporated into the liquid of the GIC [11,12]

The modification of GICs with chitosan (CS) particles may result in excessive ion release properties, leading to a reduction in pulpal cell viability. Previous cell viability assessments provided insight into the potential cellular interactions with the dental pulp. The Balb/c 3T3 embryo fibroblast cell line (3T3 cells) is used for assessments of cell viability and for assessing dental materials ISO 10993–5:2009 [13]; ISO 7405:2025 [14]. 3T3 cells are also used for resin-modified GICs [15] and have been successfully used to study the cell viability of dental composite monomers [16,17], dentine bonding agents [18,19,20] and direct pulp-cap treatment materials [21]. These cells are less sensitive to cytotoxicity than pulp fibroblasts and periodontal ligament fibroblasts [16] and have been used for GICs [22].

The primary hypotheses, to be tested by ANOVA, are as follows:

- Null Hypothesis for Material/Modification: There are no significant differences in mean cell viability among the six CS-modified material groups.
- Null Hypothesis for Time: There are no significant differences in mean cell viability across the three time points.
- Null Hypothesis for Interaction: The effect of the material/modification on cell viability is consistent across all time points. That is, the changes in viability over time for one material are not significantly different from the changes observed for another.

## 2. Materials and Methods

### 2.1. Materials and Modification

Three different commercially available hand-mix GIC materials were used in this experiment: Fuji IX GP (FN: GC Corporation, Tokyo, Japan, Batch: 1503231), Ketac Universal (KU: 3M ESPE, Seefeld, Germany, Batch: 583514), and Riva Self Cure (RSC: SDI, Bayswater, Victoria, Australia, Batch: 62657V). These GIC materials are comparable and classified under ISO 9917-1:2025, Dentistry—Water-based cements. Part 1: Powder/liquid acid–base cements [23]. The three GICs were modified in the powder phase by adding 5% or 10% (by weight (*w*/*w*%)) of a commercially available chitosan powder (CS) (Sigma-Aldrich, 448877, 3050 Spruce Street, Saint Louis, MO, USA) to the GIC [24]. After modification with CS particles, nine disc-shape GIC specimens were prepared using precise powder/liquid ratios in accordance with the manufacturer’s instructions and a previously published work [25]: (1) FN GIC powder [FN]; (2) FN GIC powder modified with 5-wt% CS particles [FN5%CS]; (3) FN10%CS; (4) KU; (5) KU5%CS; (6) KU10%CS; (7) RSC; (8) RSC5%CS and (9) RSC10%CS. For cell viability experiments, five specimens of each material (*n* = 45) were used, while four specimens of each material were used for the disc surface contact evaluation (*n* = 36).

### 2.2. 3T3 Balb/c Fibroblast Culture

Balb/c 3T3 Clone A31 fibroblasts were purchased from the National Repository for Biological Materials of South Africa, Sandingham. Working cultures were expanded from the original cryopreserved stock under standard conditions (37 °C with a 5% CO_2_ atmosphere at 95% humidity) in a sterile petri dish with a sterile DMEM high-glucose medium containing L-Glutamine and Sodium Pyruvate (HyClone, SH30243.01, GE Healthcare Sciences, Logan, UT, USA). The medium was supplemented with 10% fetal bovine serum, 1% penicillin, and 1% streptomycin (Sigma-Aldrich, P4333, 3050 Spruce Street, Saint Louis, MO, USA) in order to retain cell viability and prevent any pathogen growth. The Balb/c 3T3 fibroblasts (3T3 cells) were sub-cultured as soon as the growth phase was reached at approximately 80–90% confluence at passage 11. In order to remove the attached 3T3 cells from the bottom of the petri dish, they were trypsinized with 0.25% Trypsin-EDTA (Gibco, SV30031.01, Thermo Fisher Scientific, Waltham, MA, USA), and 100 µL of this mixture was placed into each well of a 96-well plate at a concentration of 3 × 10^4^ cells/mL. The 3T3 cells were incubated in the 96-well plate over 24 h, allowing them to attach to the well plates [26].

### 2.3. Diluent Specimen Preparation

Diluents in sterile Dulbecco’s Modified Eagle Medium (DMEM) (HyClone, GE Healthcare Life Sciences, Logan, UT, USA, Lot AD16496263) were made from various GIC and CS-modified GIC materials. For the cell viability tests, five specimens of each material (*n* = 45) were placed into a Teflon split mold (6 ± 0.1 mm height and 4 ± 0.1 mm diameter). The specimens were finished with 1200-grit wet silicon carbide paper on both sides after a 1-h bench cure and sterilized with ethylene oxide gas (Steri-Vac 4XL Gas Sterilizer, Model 400DGP, Saint Paul, MN, USA) for 24 h, including standard aeration. The sterile material specimens (after a total of 100 h following manufacture) were placed in 5 mL of sterile DMEM in sterile centrifuge tubes for three different time periods: 0–1 days, 1–7 days, and 7–21 days. Ion release from the material specimens into the DMEM occurred at 4 °C; the manufacturer’s instructions indicated that DMEM should be stored between 4 °C and 8 °C. At the end of each time period (end of day 1, 7, and 21), the centrifuge tube with diluents containing the GIC specimens was vortexed for 3 s. The GIC specimens were subsequently removed from the centrifuge tube with sterile tweezers, and the materials were placed into new sterile centrifuge tubes containing 5 mL of fresh sterile DMEM. Over the course of the specified time periods (1 day, 7 days, and 21 days), exposing the GIC to DMEM allowed for the elution of ions from the GIC into the DMEM to form the diluent. In order to perform the MTT colorimetric assay for the diluent of each material for each time period, triplicate 96-well plates per time period were used, and 300 µL of the formed GIC-diluent was divided equally into three wells per plate to assess the effect of the ions that eluted from the GIC on the 3T3 cells.

### 2.4. MTT Colorimetric and 3T3 Balb/c Fibroblast Cell Viability Assays

The 3-(4,5-dimethylthiazol-2-yl)-2,5-diphenyltetrazolium bromide (MTT) colorimetric assay measures cell viability, proliferation, and activation. It works on the principle that the amount of purple formazan formed by mitochondria is directly proportional to the number of viable cells that are still active and proliferating [27]. A 5 mg/mL MTT stock solution was prepared as per the manufacturer’s instructions (MTT, Sigma-Aldrich, M2128, 3050 Spruce Street, Saint Louis, MO, USA) in phosphate-buffered saline (Merck, D8537, 1 Friesland Drive, Longmeadow Business Estate, Modderfontein, South Africa) at pH 7.5 and was filtered through a 0.22 µ filter.

For each MTT assay, the top 300 µL of the GIC-diluents from each centrifuge tube was removed via pipette after 3 s of vortexing and was equally divided between three wells of a 96-well plate which contained 3T3 cells. No adjustments were made to the pH value before adding 100 µL of GIC-diluent to each well. It was essential to use the diluents without altering the pH because the pulp cells will be exposed to a non-adjusted pH in the teeth. The 96-well plates with 3T3 cells consisted of wells containing GIC-diluents and control wells. A total of 1500 µL of sterile DMEM was equally divided between 15 wells, with each well containing 100 µL. Each of the three 96-well plates consisted of three wells for each GIC material per analyzed day (e.g., FN, FN5%CS, and FN10%CS, KU, KU5%CS, KU10%CS, RSC, RSC5%CS and RSC10%CS on days 1, 7, and 21), as well as fifteen control wells containing DMEM. All the diluents and DMEM samples were added to the 3T3 cells once they had reached room temperature to prevent thermal shock. All of the prepared 96-well plates were incubated for 24 h to allow the diluent to have an effect on the 3T3 cells [28]. Cell viability assays were completed using MTT in accordance with an established method [28]. At the end of the 24 h incubation period, 10 µL of MTT (5 mg/mL) was added to the monolayer of cells that formed on the bottom of the plates to allow the mitochondria in the viable cells that were still active and proliferating to convert the yellow, water-soluble MTT into insoluble, dark purple formazan. After 3 h of incubation at 37 °C, 100 µL of dimethyl sulfoxide (DMSO) (Merck, Hibri-max D2650, 1 Friesland Drive, Longmeadow Business Estate, Modderfontein, South Africa) was added to each well to allow for the extraction of the purple/blue formazan crystals from the viable cells. Within 5 min of DMSO addition, measurements were recorded using a dual-wave spectrophotometer (Smart Microplate Reader, Model SMR16.1; USCN life science kit, Wuhan, China) at a test wavelength of 570 nm and a reference wavelength of 630 nm [28].

The relative viability of the 3T3 cells exposed to the diluents was calculated using the following formula: [100/(Optical density of the control 3T3 cells × Optical density of the GIC-diluent exposed 3T3 cells)]–100. This formula allowed for the results of cell viability to be presented as positive and negative percentages, indicating an increase and a decrease, respectively, in cell viability below that of the control 3T3 cells.

The pH was determined for all DMEM samples at various time intervals (Ohaus Corporation, model Starter 3100, Parsippany, NJ, USA).

### 2.5. 3T3 Balb/c Fibroblast Disk Surface Contact Evaluation

The 3T3 cells exposed to the material disc surface were evaluated based on four disc-shaped specimens (3 mm in diameter and 1 mm thick) of each material, allowing for visualization of the material’s border using inverted microscopy. The specimens were finished and sterilized as previously described. One material specimen was plated per well of a 12-well plate and was seeded with 3 × 10^4^ 3T3 cells/mL. The remainder of the well was carefully filled with DMEM on the side of the material specimen to reduce the risk of flushing the seeded cells from the material specimen’s surface. The seeding density was visually assessed for evenly distributed cells at about 80–90% confluence. Cells were quantified using a hemocytometer, and a seeding density of 3×10^4^ cells per mL was observed. This value is optimum for growth and differentiation during assessment of the material specimen. Determining the effect of the material on the morphology of the 3T3 cells in contact with the disc’s surface was the objective of this investigation. After 24 h, two specimens of each material were randomly selected for visualization, and the other two remained in the incubator to be visualized at 72 h. A light microscope (Olympus CK2 inverted microscope, Olympus Life Sciences, Center Valley, PA, USA) with a camera (Zeiss Axiocam ERc5s digital camera, Oberkochen, Germany) was used to assess the 3T3 cells at the edge of the GIC material at 10× magnification. The materials were removed from the well plates and prepared for SEM to analyze the distribution and morphology of the 3T3 cells attached to their surface at 150× and 750× magnification, respectively. The analysis of the inverted microscopy and SEM images was completed by a blinded investigator using the criteria described in a previously described analysis [29]. Cell attachment was assessed based on the presence and shape of the filopodia (cylindrical or conical in length); the lamellipodia (flat extensions or extensions elevated from the surface); and the density/translucency of the cells (low or high) [29].

### 2.6. Fixing Technique for Scanning Electron Microscopy (SEM) of 3T3 Cells

The specimens were prepared for SEM to expand upon the results obtained, especially for the RSC and KU materials. Sodium cacodylate trihydrate, also called cacodylate or cacodylic acid, was used in lieu of osmium [12,30,31].

### 2.7. Statistical Analysis

Sample size calculations were conducted based on previous results to determine the required sample size at a statistical level of *p* < 0.05 [32]. Normally distributed data were determined with the Shapiro–Wilk test. The results were analyzed with R Core Team (2013) statistical software version 3.0.1; R is a language and environment for statistical computing (R Foundation for Statistical Computing (Vienna, Austria)). The mean ± standard deviation (SD) was used to represent the digital data.

A two-way ANOVA was employed to assess the simultaneous effects of two independent factors—material/modification and time—on the dependent variable: cell viability. The model’s factors are defined as follows:

Factor A: Material/Modification with six levels: FN 5%CS, FN 10%CS, KU 5%CS, KU 10%CS, RSC 5%CS, and RSC 10%CS.
Factor B: Time with three levels: Day 1, Day 7, and Day 21. Analysis was performed with a significance level (alpha) of 0.05.

A significant *p*-value (*p* < 0.05) for any of these factors would lead to the rejection of the corresponding null hypothesis.

## 3. Results

### 3.1. Effect of Different GICs on Cell Viability

**Figure 1:**
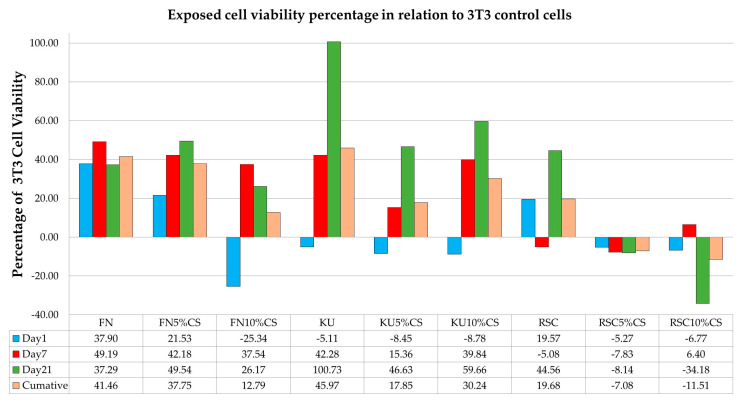
CS-modified cell viability on days 1, 7 and 21.

The two-way ANOVA indicated that the main effects of both the FN, KU and RSC materials and time are statistically significant (*p* < 0.001). More importantly, the interaction effect between material and time is also statistically significant (*p* < 0.001). The MTT assay confirmed that all materials besides KU (Day1) and RSC (Day 7) provided significant cytocompatibility and assisted 3T3 cell growth. 3T3 cells not exposed to any GIC-diluents served as the control cells, and their growth was set at 0%. The mean 3T3 cell viability following exposure to the three GIC materials—FN, KU and RSC—is shown in Table 1 and Table 2. Cell viability is shown as a percentage greater (“+”) or (“−“) less than the control 3T3 cells for each material. There was positive 3T3 cell growth at Day 1 for FN (37.9%) and RSC (19.57%), while there was a significant increase in cytocompatibility (*P* < 0.05) in 3T3 cells at day 7 for FN (49.19%) and KU (42.28%) (Figure 1). The KU-diluent on day 21 exhibited a 100.73% increase in 3T3 cell viability; this was followed by RSC (44.56%) and FN (37.29%).

Table 2 shows the significance of the CS-modified materials on days 1, 7 and 21. When compared with the 3T3 control cells’ viability, the results illustrate that the GIC-diluents of all FN materials and all CS-modified materials increased the viability of the 3T3 cells on all days (1, 7, and 21), except for FN on day 21 and FN10%CS on day 21 (Figure 1).

All the KU materials exhibited in a decrease in cell viability in relation to the 3T3 control cells for day 1. On day 7, there was an increase in cell viability, although only KU and KU10%CS presented with significantly more viable cells in comparison to the 3T3 control cells (*p* < 0.05) (Figure 1). The RSC diluents on day 1 (19.57%) exhibited the highest percentage of viable cells. Both the CS modifications exhibited a decrease in cell viability, but this was only significant for RSC10%CS (−6.77) in relation to the control 3T3 cells. On the contrary, for day 7, RSC10%CS was the only material from the RSC material group that exhibited an increase in 3T3 cell viability, and on day 21, the CS-modified RSC GICs exhibited negative cell viability in relation to the 3T3 control cells (as shown in Table 3).

### 3.2. Cell Morphology in Response to Contact with the Disc’s Surface

**Figure 2:**
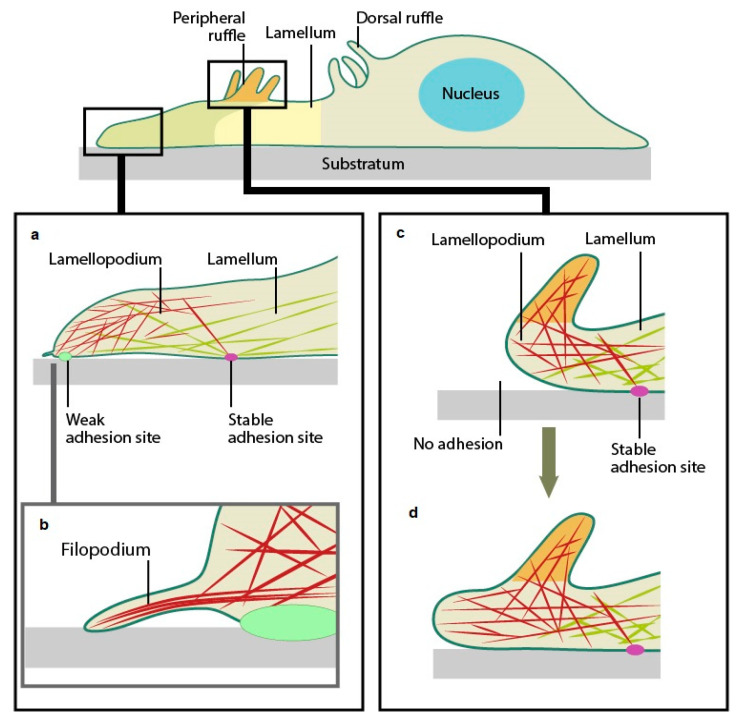
(**a**) Graphical illustration of the structural components of a 3T3 cell. (**b**) Illustration of weak and stable adhesion sites. (**c**) Illustration of the filopodium. (**d**) Illustration of no adhesion of the lamellipodium. Reprinted with permission from Ref. [33]. Copyright 2022 National University of Singapore.

The interaction of the 3T3 cells was visually assessed at the edges of the GICs at 24 and 72 h. The lamellipodium is a cytoskeletal projection and is the leading edge of the cell that is used to propel the cell forward (Figure 2b), coming from the Latin for “thin sheet” (“lamina”) and “foot” (“pod”). The filopodium is a slender and sometimes long cytoplasmic projection that serves as an extension to the lamellipodium. It provides focal adhesions between the cell and the surface of the material (Figure 2c).

**Figure 3:**
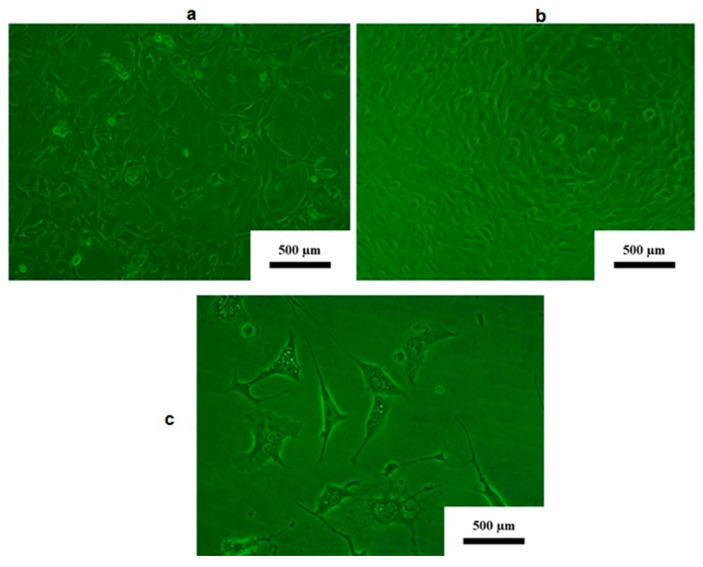
(**a**) Control 3T3 cells in this study at subconfluence. (**b**) Control 3T3 cells in this study at confluence. (**c**) Control 3T3 cells in this study at 24 h. (Light microscopy; 10× magnification.)

Figure 3a shows 3T3 cells with extensive sheet-like extensions of the lamellipodia that are in contact with other 3T3 cells. The filopodia have both cylindrical and conical extensions, and the nucleus is visible in the sheet-like as well as stellate cells. Spindle-shaped cells are also present. This figure clearly illustrates that the 3T3 cells can be stellar or spindle-shaped, depending on their movement (Figure 3a). The cells represented in Figure 3b are not a spindle or stellar shape. Once the 3T3 cells reach confluence, they become epithelioid. Figure 3c shows stellate- and spindle-shaped 3T3 cells with a visible nucleus; the lamellipodia and fibroblasts are well developed and 3−4 times the length of the 3T3 cell.

**Figure 4:**
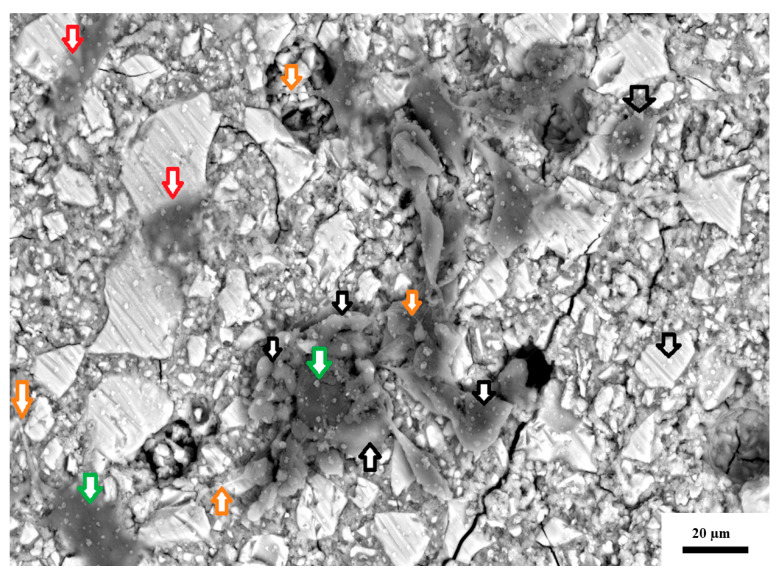
SEM image of 3T3 cells on the surface of FN at 24 h.

The green arrows in Figure 4 indicate crystal precipitation from the fixing process on the specimens for SEM analysis. This crystal precipitation did not present similarly on all specimens and varied between the GIC materials. These crystal precipitations were due to the ethanol drying process. The low crystalline contamination in FN was equally distributed between its surface and the 3T3 cells. The red arrows show cells with a lower cellular density (greater translucency), while the green arrows indicate cells with a higher cell density and the black and white arrows (Figure 4) indicate the loss of stable adhesion in the lamellum (Figure 2d). They also indicate that the lamellipodium is folded inward towards the cell nucleus. The 3T3 cell on the far right, indicated by the green arrow, is still stably adhered to the lamellum. Additionally, the orange arrow indicates filopodia, which are cylindrical and double the length of the 3T3 cell.

**Figure 5:**
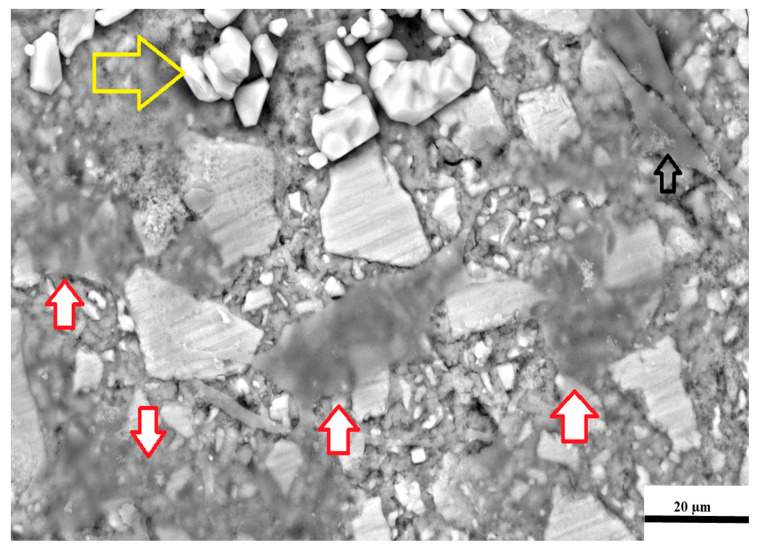
SEM image of 3T3 cells on the surface of FN at 72 h.

The yellow arrow in Figure 5 represents large crystal precipitates due to the ethanol drying process. At 72 h, the 3T3 cells (indicated by the red arrows) presented with an even lower cellular density compared to those observed in the FN SEM image at 24 h. This is due to the filler particles that can be seen through the 3T3 cells. The general shape varied between stellate (red arrows) and spindle-shaped (empty black arrow), indicating that the cells were still mobile at 72 h, with the lamellipodium presenting with filopodia that connected the 3T3 cells together. This indicates that the cells had greater viability than those at 24 h, despite their translucency. There was a loss of stable attachment present in the lamellum, and the lamellipodium was folded inward. A similarly folded lamellipodium was noted in the FN images at 24 h. The other cells were viable, with attachment, and they were either stellate- or spindle-shaped. Wide lamellipodia and filopodia up to six times the length of the 3T3 cell were present, and both high and low cell densities were noted (Figure 5).

**Figure 6:**
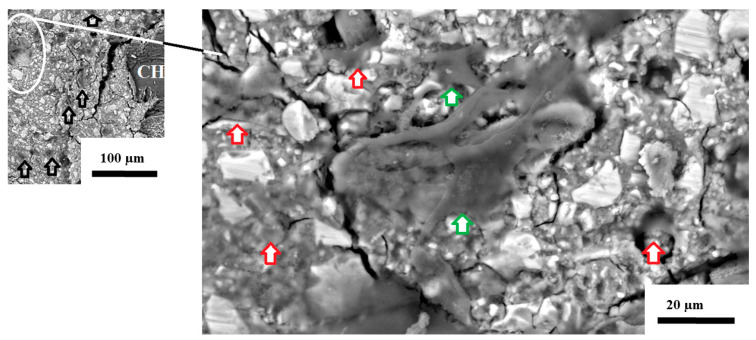
SEM image of 3T3 cells on the surface of FN5%CS at 72 h.

Figure 6 presents the CS as seen below the surface of the GIC. The expansion of the CS caused a crack in the GIC, with the empty black arrows showing that the 3T3 cells are far apart. Upon further magnification, the red arrows indicate 3T3 cells with a lower cellular density, and the green arrows indicate 3T3 cells with a higher density due to the presence of more than one 3T3 cell in that area. It appears as though the cytoplasm of the cell is widely dispersed and disturbed, and neither clear lamellipodia nor filopodia can be identified.

**Figure 7:**
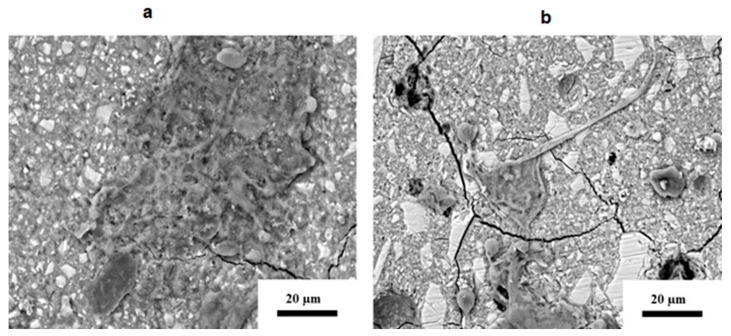
SEM image of 3T3 cells on the surface of (**a**) KU and (**b**) RSC at 24 h (displaying cell lysis).

Lysis of the 3T3 cells was observed on the surface of KU at 24 h (Figure 7a) and 72 h for all the cells in the field of view. The RSC GIC was the only material that seemed to have round extensions that resembled blebs (Figure 7b).

**Figure 8:**
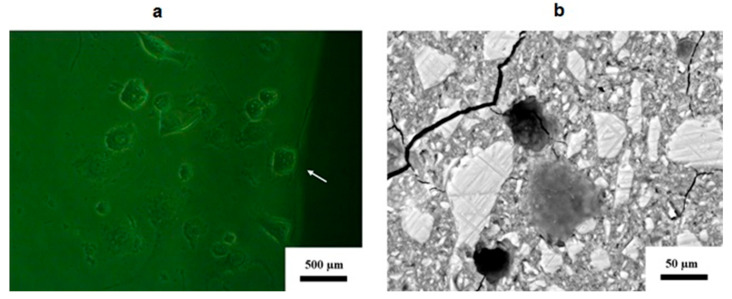
(**a**) Light microscopy image of 3T3 cells on the surface of the RSC margin at 24 h. (**b**) SEM image of 3T3 cells on the surface of RSC at 24 h, displaying no lysis.

Light microscopy images, however, showed that these extensions were the nuclei (Figure 8a). The filopodia were estimated to be twice the size of the 3T3 cell; however, due to lysis, this may not be completely accurate. Most of the 3T3 cells in Figure 8a do not exhibit lamellipodia or filopodia; however, the nuclei of all the cells were much larger than those of the control 3T3 cells. The SEM image (Figure 8b) on the surface of the GIC indicates that they have the same appearance.

Not all the cells presented with lysis (as in Figure 7b); see Figure 8b for a 3T3 cell that did not display lysis. There were, however, no lamellipodia, and very thin filopodia were present in the SEM image. This agrees well with the light microscope images for RSC, although filopodia can be seen on the 3T3 cell at the margin of the GIC (Figure 8a). Stellar 3T3 cells far away from the GIC margin presented with lamellipodia, and the filopodia were 2−3 times longer than cells themselves. The cells attached to the margin had short filopodia, and the 3T3 cells attached to the GIC presented lamellipodia as round to stellar 3T3 cells. The filopodia were the same length as the 3T3 cell or not visible at all.

## 4. Discussion

All the hypotheses were rejected since the CS-modified glass ionomers exhibited less cellular viability than the respective control materials.

The two-way ANOVA revealed statistically significant results for both the control, FN, KU and RSC materials and the time period. These findings were characterized by a highly significant material × time interaction effect. Tukey’s Honestly Significant Difference (HSD) post hoc analysis confirmed that use of the KU material resulted in cytotoxicity on day 1, and then an increased cytocompatibility was maintained from day 7 to day 21. This finding highlights a crucial difference in the long-term biocompatibility and bioactivity of the KU material compared to the other tested materials. FN was consistently biocompatible and RSC exhibited no cytocompatibility trends between the different time periods. This data suggests that KU presents potential for 3T3 cell viability applications requiring sustained cell proliferation, whereas FN will remain consistent in cell viability stimulation. For RSC, it may be difficult to predict 3T3 cell viability (Table 1).

The significant interaction effect is the most crucial finding of this analysis. The effect of time on cell growth is dependent on the material (FN, KU and RSC). While the FN graph exhibits a horizontal plateau, the graph for the RSC control exhibits a downward trajectory. The cytocompatibility of KU increases from day 7 to 21, and this represents a disordinal interaction—the material vs. time interaction changes the direction of the cytocompatibility (Figure 1).

In an in vivo study on rats, using FN produced moderate pulpal responses at day 8. The results at day 30 indicated no harmful effects, since reparative dentine formed, similar to the control group [34]. This in vivo result matches the results of the present study, as the viability of the 3T3 cells exposed to FN-diluents was greater on days 1, 7, and 21.

The visual representation of the cells via SEM analysis reinforces the quantitative results of the MTT assay. The decline in cell viability seen on day 1 for KU and day 7 for RSC is visually confirmed in the SEM images, where there is cell breakdown and poor cell adhesion to the surface of the KU and RSC CS-modified GIC. GICs are known to release ions in a burst in the early stages after placement. These initial cytotoxic effects are potent, especially for CS-modified materials due to the high ion release [24], and have been visually confirmed to cause physical damage to the 3T3 cells. The release of ions into the DMEM used for the 3T3 cells disrupts normal cellular processes and leads to damage in the cells. Sodium and free fluoride are more readily released from KU, RSC and their CS modifications compared to FN [24]. Sodium is released as the counter ion for fluoride when the fluoride in the GIC moves into the diluent [24]. As part of this process, the hydroxide from the diluent moves to the GIC. Sodium also disrupts the cross-links in the polysalt matrix, also facilitating ion exchange between the GIC and the hydroxide in the diluents [35]. The more hydroxyl ions that move into the GIC, the lower the pH of the diluent medium [24,36]. The initial DMEM pH of 7 progressively increased and then stabilized, with a very small variance in the pH between the materials and between the specified time intervals (1, 7, and 21 days) for the control materials (Table 2). Comparing Figure 1 and Table 2, there is no obvious visible correlation between the pH and the cytocompatibility percentages. With pH playing a role in cell viability, it is important to consider that various ion combinations could result in a similar pH, yet provide a more toxic or biocompatible environment for the cells. The conclusion is that the consistently reduced release of ions [24] from FN and FN5%CS groups maintained a higher level of cell viability throughout the study. The cells exhibited higher growth and attachment in the SEM images, with the fibroblasts forming a more cohesive layer. Modifying GICs with commercially available large-particle CS led to FN having a superior initial biocompatibility compared to KU and RSC for all three time periods.

FN and its CS modifications exhibited a wide range of pH values compared to KU, RSC and their respective CS modifications. Similar results of an increase in fluoride release as the CS percentage increased [37] were observed, confirming that monovalent ions increase fluoride release [38] and additionally suggesting that most of the fluoride is present in crystalline form and therefore not available for release.

The amount of fluoride released was directly proportional to the fluoride content for manufacturer-produced GIC, indicating that increasing the percentage of CS results in faster fluoride release [37]. This is only true if the fluoride is not part of the crystalline inclusions. When the fluoride is located within the crystalline inclusions, it is largely unavailable after the acid–base reaction is fully completed [39]. Therefore, as there is a decreased release of fluoride and various other ions [24] over the various time periods, the cell viability will also increase above that of the 3T3 control cells. There was however one material (RSC10%CS) that retained a decrease in cell viability on day 21. In vitro, cytocompatibility does not merely refer to cell viability; the direct effect on cells should also be considered. The adhesion and spread of the 3T3 cells on the surface of the materials also provide insights into how these cells react with the material. It has been suggested that cellular function influences cellular attachment and could thus provide insight into cytotoxicity [40,41] and therefore cytocompatibility. The morphology of the 3T3 cells in the present study was consistent with that found in other studies [42,43]. 3T3 cells attach to the material substrate by the lamellipodium at two sites (Figure 2). Additionally, all light microscopy images indicated that the 3T3 cells were attached to all GIC materials via filopodia, although they generally were not always stellar and spindle-shaped, but rounder, as was the case with the RSC GICs (Figure 7 and Figure 8).

A cell viability study with resin-modified glass ionomer cement (RMGIC) materials concluded that round cells, with short cellular processes originating from their cytoplasmic membrane (lamellipodia and filopodia), seemed to progress towards cell death, apparently caused by the disruption of their plasma membrane [43]. In our study, a similar result was confirmed based on the morphology of the 3T3 cells exposed to the RSC and KU GICs due to the low cell viability at 24 h (Figure 1). The 3T3 cell viability was especially low on day 1, and the SEM images (Figure 7) indicated that the 3T3 cells had severe lysis of the cytoplasm. The low cell viability persisted for RSC diluents on day 7, but a positive viability of 3T3 cells was observed for KU. It has been postulated that, compared to various RMGICs, the release of ions like aluminum, fluoride, strontium, and zinc from FN did not contribute to the decrease in cell viability as much as it did for the unreacted resin monomers [43]. This hypothesis highlights how composite methacrylate monomer resins (Z100, 3M ESPE, Seefled, Germany) and RMGICs (Vitremer and Vitrebond, 3M ESPE, Seefeld, Germany) dissolve the cell membrane as it is incorporated into the lipid bilayers [44]. It was concluded that the cytocompatibility effects from GICs could be attributed to the aluminum and/or iron present in the diluent and the oxidative stress that it causes within the cells, considering the high concentration of aluminum released from various GICs. The CS-modified KU and RSC materials also release up to 300% higher levels of aluminum and strontium than the commercial materials [24]. The amount of aluminum released after 24 h is the highest for KU control materials, followed by RSC and then FN [24]. This sequence of release also matches the cell viability sequence (Table 1). Other released ions, such as fluoride, sodium, silicon, and strontium, therefore all play a role in the ionic strength of the diluent. A study with FN found no correlation between the reduction in 3T3 cell viability and the concentration of fluoride released by FN after 24 h [28]. FN showed a decrease in cellular viability; however, one study found no difference in the expression of IL-1α, IL-6, and 18S rNA genes after 24 h between the two materials [45]. The lanthanum used in KU as a radiopacifier is also a phosphate binder.

Additionally, lanthanum has also been noted to displace calcium due to the deposition of lanthanum-phosphate in teeth [46]. Aluminum and lanthanum can change the electrical properties of a membrane once they are adsorbed onto the phosphatidylcholine liposomes; the extent of this influence is dependent upon the pH [47]. Although the electrical activity of the 3T3 cell membrane and the zeta potential of the diluents and dental materials were not assessed in this study, a conclusion could be drawn about the effect of lanthanum on the 3T3 cells based on its concentration at day 1 for KU and the CS-modified materials. Lanthanum is able to stimulate mitogenicity at low concentrations of 0.00003599 ppm [48]. The concentration of lanthanum released into de-ionized water from the KU specimens is as follows: KU (0.000261 ppm), KU5%CS (0.01048 ppm), and KU10%CS (0.015138 ppm) [24]. Studies have assessed the effect of a lanthanum concentration of 0.0007198 ppm on 3T3 cells, showing apoptotic characteristics, namely perinuclear chromatin condensation and fragmentation of the cell nucleus [49]. In this study, the lowest lanthanum concentration released from KU into de-ionized water was 0.000261 ppm on day 1. The magnitude of lanthanum released from KU, as well as the CS-modified KU materials, on day 1 explains the lower cell viability, which was below that of the control 3T3 cells, of the diluents on day 1. Although lanthanum release was not assessed on days 7 and 21, it could be deduced that the lanthanum values dropped to well below that of KU (0.000261 ppm) on day 15. This deduction regarding the release of lanthanum into the GIC-diluents on days 7 and 21 is plausible, as if was not the case, then the ppm concentration would not be low enough to stimulate the mitogenic effect, indicated by the surging cell viability in Table 1 and Table 2 for KU on days 7 and 21. CS-modified RSC continued to decrease the viability of 3T3 cells well below that of the control on day 21. An antibacterial study with *S. mutans* found that after 24 h of exposure to *S. mutans*, CS-modified RSC GICs were structurally more resilient to the environment than CS-modified FN and KU GICs [50]. Chitosan has a high affinity for proteins in the form of amino acids, and these are provided in the DMEM due to the presence of L-Glutamine, an amino acid. The surface of CS-modified GICs in powder form presents with multiple large CS particles [50]. These large and exposed CS particles degrade in DMEM, increasing the exposure of the GIC surface to the DMEM diluent. On human gingival fibroblasts, a 2% CS-modified FN performed the best with regard to cell viability—assessed by cytocompatibility—when the CS was used in liquid form, rather than as CS particles, as used in this study. The use of CS liquid presents an improved handling protocol for GIC materials [51]. A limitation of this study on the Disc Surface Contact of materials to 3T3 cells is that exposure to moisture plays an important role in GIC maturation and final matrix formation. The material samples released ions into the DMEM at 4 °C, but there also could be a decrease in ion release compared to that at temperatures between 21% and 37 °C [52]. 3T3 cells are considered to be more sensitive to cytotoxic challenge compared to human pulp fibroblasts, and future studies could assess GIC-diluents on this cell line.

## 5. Conclusions

This study’s most significant finding is the clear difference in biocompatibility among the tested GIC control materials, especially during the critical initial stages. The sustained, high cell viability observed with FN, in contrast with the initial cytotoxic effects of RSC and KU DMEM diluents, provides crucial biological evidence for material selection in clinical practice. Clinicians should therefore ensure hard-setting calcium hydroxide pulpal protection in dentine bridges less than 1mm in thickness. The structural integrity and the lamellipodia presenting with filopodia also varied between specimens. The KU material appears to actively support a sustained increase in cell proliferation, while FN exhibited a consistent plateau, illustrating a favorable long-term biocompatibility profile. The MTT cell viability tests using 3T3 cells indicated that there was an increase in cell viability from 1 to 21 days for FN and CS-modified materials. Regarding CS-modified FN materials, the 5% CS material exhibited a slightly higher mean viability at day 21 compared to the 10% CS material, although this difference may not be statistically significant. This finding suggests that for FN, the 5% CS-modified material has a similar effect to the control material.

A similar trend was observed for CS-modified RSC materials, with a decrease in cell viability for the 5 and 10% CS modifications on day 21. The 5% CS material maintained a negative cell viability from day 1 to day 21, while the 10% CS material resulted in a statistically greater cytotoxicity; thus, RSC should not be modified with 5% or 10% CS due to cytotoxicity.

In contrast, the CS-modified KU materials showed a more favorable response once the day 1 cytotoxicity had passed. On days 7 and 21, KU 5%CS and KU 10%CS exhibited an increase in cytocompatibility. This finding indicates that for KU, CS modification is viable only at 10%. CS-modified FN and KU materials can be used as temporary restorations, including for carious lesion re-mineralization with a dentine bridge of more than 1mm, e.g., for two-step caries removal procedures where re-entry is required. Based on the literature, using liquid CS remains the most appropriate method to incorporate CS into GIC.

## Figures and Tables

**Table 1:** Cell viability of the materials in relation to the 3T3 control cells.

Comparison between Groups and Time	Mean Difference	Standard Error	95% Confidence Interval	Adjusted *p*-Value
KU Day 21 vs. FN Day 21	0.164	0.044	(0.076, 0.252)	<0.001
KU Day 21 vs. RSC Day 21	0.154	0.044	(0.066, 0.242)	<0.001
FN Day 1 vs. FN Day 21	0.311	0.044	(0.223, 0.399)	<0.001
RSC Day 1 vs. RSC Day 21	0.204	0.044	(0.116, 0.292)	<0.001
KU Day 1 vs. KU Day 21	−0.052	0.044	(−0.140, 0.036)	0.540
**Day(s)**	**Material**			
**Comparison within groups**	**FN**	**KU**	**RSC**	
**Day 1 3T3 cells**	37.90 *	−5.11	19.57 ^	
**Day 7 3T3 cells**	49.19 *	42.28 *	−5.08	
**Day 21 3T3 cells**	37.29 ^	100.73 *	44.56 *	

(*): Indicates a significant difference (*p* < 0.01) in 3T3 cell viability percentage. (^): Indicates a significant difference (*p* < 0.05) in 3T3 cell viability percentage.

**Table 2:** Cell viability for FN, KU and RSC materials and their respective CS modifications in relation to the 3T3 control fibroblasts.

Day	Material		
	FN	FN5%CS	FN10%CS
**Day 1 3T3 cells**	37.90 *	21.53 ^	−25.34 ^
**Day 7 3T3 cells**	49.19 *	42.18 *	37.54 *
**Day 21 3T3 cells**	37.29	49.54 ^	26.17
	**KU**	**KU5%CS**	**KU10%CS**
**Day 1 3T3 cells**	−5.11	−8.45	−8.78 *
**Day 7 3T3 cells**	42.28 *	15.36	39.84 *
**Day 21 3T3 cells**	100.73 *	46.63 *	59.66 *
	**RSC**	**RSC5%CS**	**RSC10%CS**
**Day 1 3T3 cells**	19.57	−5.27	−6.77 ^
**Day 7 3T3 cells**	−5.08	−7.83 ^	6.40
**Day 21 3T3 cells**	44.56 *	−8.14	−34.18 ^

(*): Indicates a significant difference (*p* < 0.01) in 3T3 cell viability percentage. (^): Indicates a significant difference (*p* < 0.05) in 3T3 cell viability percentage.

**Table 3:** pH of DMEM samples for FN, KU and RSC materials and their respective CS modifications in relation to the 3T3 control fibroblasts.

Day	Material		
	FN	FN5%CS	FN10%CS
**Day 1 DMEM pH**	8.93	8.85	8.88
**Day 7 DMEM pH**	8.69	8.65	8.71
**Day 21 DMEM pH**	8.29	8.41	8.78
	**KU**	**KU5%CS**	**KU10%CS**
**Day 1 DMEM pH**	8.9	8.8	8.84
**Day 7 DMEM pH**	8.91	8.65	8.41
**Day 21 DMEM pH**	8.84	7.77	8.03
	**RSC**	**RSC5%CS**	**RSC10%CS**
**Day 1 DMEM pH**	8.71	8.89	9.02
**Day 7 DMEM pH**	8.76	8.88	8.77
**Day 21 DMEM pH**	8.93	8.87	8.93
**DMEM pH**	7		

## Data Availability

The original contributions presented in this study are included in the article. Further inquiries can be directed to the corresponding author.

## Abbreviations

The following abbreviations were used in the manuscript:

GIC	glass ionomer cement
CS	Chitosan
CS-modified	chitosan-modified
FN	Fuji IX GP
KU	Ketac Universal
RSC	Riva Self Cure
*S. mutans*	Streptococcus Mutans Species
